# Upcycling waste apples into platform chemicals: pilot-scale production of lactic and succinic acids

**DOI:** 10.1186/s13068-026-02807-w

**Published:** 2026-09-07

**Authors:** Laís Portugal Rios da Costa Pereira, Roland Schneider, Agata Olszewska-Widdrat, Barbara Sturm, Korbinian Kaetzl

**Affiliations:** 1 Section of Grassland Science and Renewable Plant Resources, Faculty of Organic Agricultural Sciences, University of Kassel, Steinstrasse 19, 37213 Witzenhausen, Germany; 2 Department of Microbiome Biotechnology, Leibniz Institute for Agricultural Engineering and Bioeconomy, Max-Eyth-Allee 100, 14469 Potsdam, Germany; 3 Albrecht Daniel Thaer-Institute of Agricultural and Horticultural Sciences, Humboldt University of Berlin, Lentzeallee 55-57, 14195 Berlin, Germany; 4 Chair of Urban Water Management, Institute of Urban and Industrial Water Management, Dresden University of Technology, Bergstrasse 66, 01069 Dresden, Germany

**Keywords:** Fermentation, Lactic acid, Pilot scale, Succinic acid, Waste to value

## Abstract

The conversion of renewable biological resources into value-added products is essential for supporting the transition to a sustainable bioeconomy. This study explores the use of waste apples—a by-product generated in large quantities along the fresh apple supply chain—as feedstock for the production of two industrially relevant platform chemicals: lactic acid (LA) and succinic acid (SA). A screening identified *Heyndrickxia coagulans* A203 and *Actinobacillus succinogenes* DSM 22257 as the most suitable strains for LA and SA production from apple mash, the sugar-rich fraction obtained by pressing waste apples (excluding the solid peel and seed residues). A simplified procedure, omitting costly centrifugation and sterilization steps between enzymatic liquefaction and fermentation, was successfully scaled up, resulting in 73.8 g L^−1^ LA with a yield of 0.91 g g^−1^ and 36.8 g L^−1^ SA with a yield of 0.69 g g^−1^. The results demonstrate the potential of waste apples as a low-cost, effective feedstock for the biotechnological production of high-value platform chemicals, contributing to the valorization of agri-food waste within a sustainable and circular bioeconomy.

**Supplementary Information:**

The online version contains supplementary material available at https://doi.org/10.1186/s13068-026-02807-w.

## Background

The transition toward a circular bioeconomy necessitates the development of innovative strategies aimed at minimizing waste and enabling the conversion of residual biomass into value-added products [1]. In response to this global challenge, numerous initiatives promoting circularity and sustainability have been implemented globally. Among these, the Bioeconomy Strategy of the European Union (EU) is notable for supporting the use of biowaste—including food and agricultural residues—as feedstock for the production of biobased products such as platform chemicals, biomaterials, and bioenergy [2]. Within this context, the valorization of agri-food waste into high-value biobased compounds represents a viable approach to waste reduction and sustainable resource management [3, 4].

Among the various types of agri-food waste, surplus and discarded apples—a by-product generated throughout the fresh apple supply chain—represent promising feedstock due to their availability and biochemical composition [5]. The EU, one of the world’s leading apple producers, harvested 11.5 million tons in 2023 [6], a substantial quantity that also generates significant waste. In fact, estimates indicate losses of 20.3% in primary production (pre- and postharvest), 1.6% in processing and manufacturing, 9.6% in distribution, and 17.7% in consumption [7]. Once these apples are no longer suitable for food or feed use due to safety, quality, or technical constraints and in line with the cascade use principle outlined in the EU Waste Framework Directive [8], they can be redirected to the production of platform chemicals, presenting a valuable opportunity for resource recovery within the framework of biorefineries [3, 9].

Platform chemicals are significant because of their versatility as intermediates that can be transformed into valuable compounds across multiple industries [10]. In particular, lactic acid (LA) and succinic acid (SA) have diverse applications in the food and beverage, pharmaceutical, and cosmetic sectors. Moreover, their use as monomers in the production of polylactic acid (PLA) and polybutylene succinate (PBS) has attracted increasing interest, as these bioplastics offer environmentally friendly alternatives to fossil-based materials [11, 12].

Owing to its content of fermentable sugars and nutrients [5], apple-derived feedstock represents a compelling substrate for fermentation processes aimed at the production of LA and SA. LA production has focused primarily on the use of apple pomace—the solid residue remaining after juice extraction—at the laboratory scale under batch and repeated-batch operation modes [13, 14], whereas SA production has focused mainly on process modeling and technoeconomic analyses related to the utilization of this by-product [15, 16]. These studies provide an initial assessment of the potential of apple pomace as a substrate for LA and SA production; however, research on pilot-scale processes involving waste apples is currently nonexistent. Although pilot-scale studies have been conducted with other types of food waste [17–20], no such studies have been carried out on waste apples, highlighting a critical gap in the development of strategies for repurposing this residual biomass. This gap is particularly relevant given the potential of waste apples as feedstock, which offer advantages over the extensively studied apple pomace by offering readily fermentable substrate and requiring minimal pretreatment.

The efficiency of fermentation processes depends not only on the substrate but also the selection of appropriate microbial strains. *Heyndrickxia coagulans* (formerly *Bacillus coagulans*) is commonly used for LA production due to its high yields and productivities. This microorganism has been evaluated on a variety of substrates and has demonstrated the ability to ferment both C5 and C6 sugars and to tolerate a broad range of inhibitory compounds that are commonly present in biomass-derived media [14, 21, 22]. Additionally, its ability to operate at elevated temperatures reduces the risk of contamination, further enhancing its suitability for industrial applications [20]. With respect to SA production, compared with other strains, such as *Basfia succiniproducens*, *Actinobacillus succinogenes* has emerged as a promising candidate due to its ability to convert both pentoses and hexoses into SA and achieve higher yields [23, 24].

Based on the above background, this study investigates the use of waste apples as feedstock for the pilot-scale production of LA and SA. A screening of LA- and SA-producing bacteria was performed to identify the most efficient strain for converting waste apples into these organic acids. A simplified and less energy-intensive process employing the selected strains (*H. coagulans* A203 and *A. succinogenes* DSM 22257) was effectively scaled up, representing an innovative approach to exploring and establishing the pilot-scale production of LA and SA from waste apples while providing a comprehensive account of the entire process.

## Methods

### Raw material and preparation

Jonagold apples (*Malus domestica* ‘Jonagold’), purchased from Werder Frucht GmbH (Groß Kreutz, Germany), were provided by the Department of System Process Engineering at the Leibniz Institute for Agricultural Engineering and Bioeconomy (ATB, Potsdam, Germany) after they reached conditions unsuitable for consumption (Figure S1a). A portion of the apples had already undergone natural fermentation, and others showed visible mechanical damage. In addition, signs of insect infestation were detected, further compromising their suitability for direct consumption. The apples were pressed using a screw press (Type Cv, VETTER Maschinenfabrik GmbH & Co. KG, Kassel, Germany), with a sieve pore size of 0.8 mm and a gap width of 10 mm. The resulting apple mash (AM) (Figure S1b) contained 71.6 ± 1.5 g L^−1^ fructose, 21.5 ± 0.7 g L^−1^ glucose, and 7.5 ± 0.3 g L^−1^ sucrose and was frozen at −20 °C until further use. The solid fraction, composed mainly of peel and seeds, was not evaluated in this study.

### Enzymatic treatment of apple mash

#### Screening of enzymes

To increase the fermentable sugar content in the AM and reduce its viscosity, the enzymes Pectinase L40 (ASA Spezialenzyme GmbH, Wolfenbüttel, Germany), Cellic CTec3 HS, Dextrozyme GA and Viscoferm (Novozymes A/S, Bagsvaerd, Denmark) were separately evaluated. The experiments were carried out in duplicate in shaking flasks containing 50 g of AM and incubated at the original pH (4.5), 50 °C and 150 rpm for 24 h. The enzyme dosage was 1.0 mL_e_ kg_bm_^−1^. The combination of Pectinase L40 and Cellic CTec3 HS was also evaluated under the same conditions at two levels: high loading (1.0 mL_e_ kg_bm_^−1^ each) and low loading (0.5 mL_e_ kg_bm_^−1^ each). A control experiment without the addition of enzymes was included for comparison.

#### Enzymatic liquefaction of apple mash for microorganism screening

After the most effective enzymes and their optimal concentrations were selected, the AM was enzymatically liquefied for subsequent use as a medium for microorganism screening. AM (1.0 or 2.0 kg) was transferred to a sterilized bioreactor (Biostat Bplus, Sartorius AG, Göttingen, Germany), where the enzymes Cellic CTec3 HS and Pectinase L40 were added at a concentration of 0.5 mL_e_ kg_bm_^−1^ each. The reaction was carried out at the original pH (4.5), 50 °C and 100 rpm for 24 h, after which the resulting medium was centrifuged at 5000 rpm for 15 min to remove solid particles.

This process was repeated using 5.6 kg of AM to generate sufficient medium for inoculum cultivation in the prepilot validation tests and pilot-scale fermentations.

### Screening of microorganisms

To evaluate whether AM can support microbial growth and to identify the most adaptable LA- and SA-producing strains, screenings were conducted.

For LA production, *Heyndrickxia coagulans* strains A35, A107, A138, A203, and A559, which were all isolated at ATB and preserved in its internal collection, were selected from 700 strains based on their ability to consume sucrose and their performance in previous studies [20, 21]. The strains were reactivated from cryostock in 5 mL of MRS broth (Merck, Darmstadt, Germany) at 50 °C for 16–18 h.

For SA production, all available SA-producing strains, *Actinobacillus succinogenes* DSM 22257 and *Basfia succiniproducens* DSM 22022, CCUG 57762, CCUG 57763, CCUG 57764, CCUG 57765, and CCUG 57766, obtained from the Leibniz Institute DSMZ—German Collection of Microorganisms and Cell Cultures GmbH (Braunschweig, Germany) and the Culture Collection University of Gothenburg (Gothenburg, Sweden), were tested. Reactivation from the cryostock was performed in 5 mL of tryptic soy broth (TSB) (Merck, Darmstadt, Germany) at 37 °C for 16–18 h.

Microorganism screening was conducted in duplicate in a Bioscreen C (Norden Logic Oy, Helsinki, Finland) optical density (OD) meter using AM supernatant (Sect. "Enzymatic liquefaction of apple mash for microorganism screening"), both with and without supplementation of 5 g L^−1^ yeast extract (Ohly GmbH, Deutsche Hefewerke GmbH & Co. OHG, Hamburg, Germany). MRS broth served as the control for LA producers, whereas nutrient broth (Merck, Darmstadt, Germany) was used as the control for SA producers. AM-based medium was adjusted to a pH of 6.5–7.0 with 20% v/v NaOH and subsequently sterile-filtered using a 0.2 µm filter, while the control medium was autoclaved following the manufacturer’s instructions. Measurements were taken automatically every 5 min for 24 h in 250 µL of medium with 20 µL of inoculum. The incubation temperature was set to 50 °C for LA bacteria and 37 °C for SA bacteria.

The best-performing strains were selected for screening in bioreactors.

### Screening of microorganisms in bioreactors

#### Inoculum preparation

*H. coagulans* strains A35, A138, and A203 were used in LA fermentations. Inocula were cultivated in 60 mL of MRS broth with 0.67 g of Everzit Dol dolomite (Evers, Hopsten, Germany) as a buffer at 40 °C and 100 rpm for 15 h.

For SA fermentations, *A. succinogenes* DSM 22257 was initially grown in 25 mL of TSB at 37 °C and 150 rpm for 20 h.

#### Fermentation

Fermentations were performed in duplicate in 0.25 L working-volume bioreactors (EloFerm Duet, EloSystems GbR, Berlin, Germany).

LA fermentations were carried out in 0.25 L of nonsterile AM supernatant (Sect. "Enzymatic liquefaction of apple mash for microorganism screening") supplemented with 10 g L^−1^ powdered yeast extract and inoculated at 2% (v/v). The yeast extract concentration was increased relative to the OD-based microorganism screening (Sect. "Screening of microorganisms") to ensure adequate nutrient availability for the strain during fermentation, compensating for the high sugar content in the AM. The temperature was maintained at 50 °C, and the pH was controlled at 6.0 using 20% v/v NaOH with a stirring speed of 200 rpm. The most efficient strain was further evaluated under identical conditions in the absence of yeast extract supplementation.

SA fermentations were conducted using 0.20 L of sterilized (121 °C for 15 min) AM supernatant (Sect. "Enzymatic liquefaction of apple mash for microorganism screening") supplemented with 10 g L^−1^ of yeast extract and 1 g L^−1^ of NaHCO_3_ (VWR Chemicals, Darmstadt, Germany), added as a 25 mL sterile solution. The inoculum was added at 10% v/v, and fermentations occurred at 37 °C, with the pH maintained at 6.7 using 20% w/v Na_2_CO_3_ at a stirring speed of 200 rpm. A different setup for SA fermentation was required to meet the metabolic needs of the microorganism, which included a carbon source to support carbon fixation and sterile conditions due to its lower optimal growth temperature. Additionally, preliminary tests (unpublished data) indicated a limitation in cell performance at higher sugar concentrations, which further justified the use of smaller quantities of AM and its subsequent dilution. SA fermentations without yeast extract supplementation were also conducted under the same conditions detailed previously.

### Prepilot-scale validation test

Following strain selection, a simplified procedure was tested in duplicate in 1.0 L working-volume bioreactors (Biostat Bplus, Sartorius AG, Goettingen, Germany) prior to implementation at the pilot scale. Unlike the approaches described in Sects. "Enzymatic liquefaction of apple mash for microorganism screening" and "Fermentation", enzymatic liquefaction and fermentation were sequentially performed in the same bioreactor, without centrifugation or sterilization (for SA) of the medium prior to fermentation. Inoculum and fermentation procedures were conducted as follows.

#### Inoculum preparation

To serve as the preculture for LA fermentations, *H. coagulans* A203 was cultivated under sterile conditions for 15 h in a 0.2 L working-volume bioreactor (EloFerm Duet, EloSystems GbR, Berlin, Germany) using 160 mL AM supernatant (Sect. "Enzymatic liquefaction of apple mash for microorganism screening") and 10 g L^−1^ yeast extract supplied as a 40 mL solution under the same conditions detailed in Sect. "Fermentation".

*A. succinogenes* DSM 22257 was grown for 16 h in 0.25 L working-volume bioreactors, as outlined in Sect. "Fermentation".

#### Enzymatic liquefaction and fermentation

For LA fermentations, 1.0 kg of AM underwent enzymatic liquefaction under the conditions described in Sect. "Enzymatic liquefaction of apple mash for microorganism screening". After 24 h, the medium was directly supplemented with 10 g L^−1^ powdered yeast extract, and fermentation was initiated at the temperature, pH, stirring speed, and inoculum concentration conditions specified in Sect. "Fermentation".

SA fermentations were conducted with 0.8 kg of AM. Following enzymatic liquefaction, the medium was heated to 85 °C for 15 min to inhibit the growth of competing microorganisms at a mild fermentation temperature (37 °C). The medium was then supplemented with 10 g L^−1^ yeast extract and 1 g L^−1^ NaHCO_3_, added as a 0.1 L sterile solution. The fermentation parameters were consistent with those detailed in Sect. "Fermentation".

### Process scale-up

The scaling-up of the process was carried out in a Biostat UD bioreactor (B. Braun Biotech International, Germany) for both LA and SA fermentations, each performed in duplicate. Compared with the laboratory-scale protocol (Sect. "Prepilot-scale validation test"), the pilot-scale procedure required modifications to handle the increased working volume. The overall strategy remained consistent with the laboratory-scale protocol presented in Sect. "Prepilot-scale validation test" and is summarized below. However, the inocula were prepared in larger bioreactors to ensure sufficient cell density, and medium handling required careful monitoring to maintain homogeneity and prevent contamination.

#### Inoculum preparation

For LA pilot-scale fermentations, *H. coagulans* A203 was cultivated for 15 h in a 0.5 L working-volume bioreactor (EloFerm Duet, EloSystems GbR, Berlin, Germany), as described in Sect. "Inoculum preparation".

Inocula for SA fermentations were also prepared following the procedure in Sect. "Inoculum preparation", with the distinction that cultivation was carried out in a 2.1 L working-volume bioreactor (Biostat Bplus, Sartorius AG, Goettingen, Germany).

#### Enzymatic liquefaction and fermentation

During the pilot-scale experiments, a total of 30.0 kg of AM was used for 30.0 L of LA fermentation, and 20.0 kg of AM was used for 25.0 L of SA fermentation. The procedure outlined in Sect. "Enzymatic liquefaction and fermentation", comprising enzymatic liquefaction, inactivation (for SA), and fermentation, was strictly followed, along with the corresponding fermentation parameters.

### Sampling and termination of fermentations

For each fermentation described in Sects. "Fermentation", "Enzymatic liquefaction and fermentation", and "Enzymatic liquefaction and fermentation", one sample was collected at 2-h intervals, inactivated at 95 °C for 20 min, and stored at −20 °C for subsequent quantification of sugars and organic acids. Fermentations were terminated when base consumption ceased.

### Analytical methods

Sugar and organic acid concentrations were determined by high-performance liquid chromatography (HPLC) using an Azura system (Knauer, Berlin, Germany) equipped with an Azura RID 2.1L refractive index detector (Knauer, Berlin, Germany) and a Eurokat H column (300 mm × 8 mm, Knauer, Germany). The mobile phase was 0.01 N H_2_SO_4_ at a flow rate of 0.8 mL min^−1^. LA optical purity was determined using the same HPLC system and a Chirex 3126 column (150 mm × 4.6 mm, Phenomenex, Aschaffenburg, Germany) with 2 mM CuSO_4_ as the mobile phase at a flow rate of 1.0 mL min^−1^. All HPLC analyses were performed in duplicate. Yield was calculated as grams of product formed per gram of sugar consumed, and productivity was calculated based on the end of base consumption. Statistical analyses were performed using Microsoft Excel. For comparisons between two group means, Student’s t-test was performed. For comparisons among three or more group means, one-way analysis of variance (ANOVA) followed by Tukey’s test was used to identify significant differences between groups.

## Results and discussion

### Enzymatic treatment of apple mash

Although enzymes are widely used in bioconversion processes to enhance overall performance, for instance, by increasing the availability of fermentable sugars [20, 21] or degrading inhibitory compounds [25], in the case of AM, the application of various enzymes did not result in a significant increase in total reducing sugars (TRS) compared with the control (*p*>0.05), as shown in Table 1. This outcome is likely due to the absence of starch, which is typically converted into glucose, fructose and sucrose during apple ripening [26], as well as the minimal levels of cellulose and hemicellulose in the dry matter, which was also relatively low (11.1 ± 0.0%). Consequently, the amylase-based preparation Dextrozyme GA, along with cellulase- and hemicellulase-based enzyme cocktails Cellic CTec3 HS and Viscoferm, had limited substrate available for hydrolysis.

**Table 1:** Total reducing sugars (TRS) after the enzymatic treatment of the apple mash

Enzyme	TRS (g L^−1^)
No enzyme (control)	95.7 ± 0.3^a^
Pectinase L40	95.7 ± 1.1^a^
Dextrozyme GA	97.4 ± 2.3^a^
Cellic CTec3 HS	95.6 ± 1.9^a^
Viscoferm	94.1 ± 1.6^a^
Cellic CTec3 HS + Pectinase L40 (high loading)	95.5 ± 0.7^a^
Cellic CTec3 HS + Pectinase L40 (low loading)	96.1 ± 1.4^a^

The values represent the mean ± standard deviation. Different letters indicate statistically significant differences between means according to Tukey’s test (p < 0.05)

Whereas the TRS remained unchanged (Table 1), a considerable reduction in viscosity was observed when Cellic CTec3 HS was evaluated individually. High viscosity represents a disadvantage in fermentation medium, as it can impair mass transfer, leading to gradients and unequal cell exposure to optimal conditions, thereby reducing process efficiency [20]. Moreover, elevated viscosity can hinder downstream processing (DSP), particularly separation operations such as filtration, resulting in lower yields [27]. Although Pectinase L40 reduced viscosity to a lesser extent, its inclusion was still considered relevant, as pectinases are commonly used in fruit mash processing to improve yields and reduce viscosity [28]. Because no difference in medium viscosity was observed between the higher and lower enzyme concentrations, the combination of Pectinase L40 and Cellic CTec3 HS at a low loading (0.5 mL_e_ kg_bm_^−1^) was selected for the enzymatic liquefaction of the AM.

### Screening of microorganisms

**Fig. 1:**
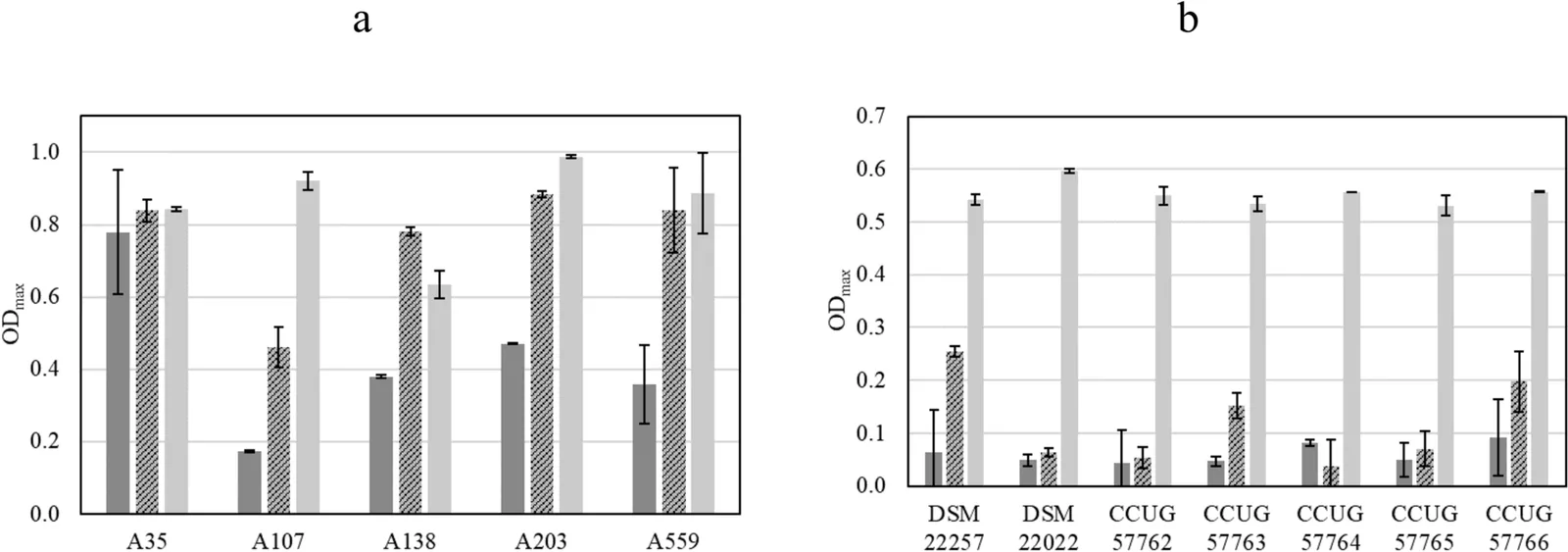
Growth of lactic acid- and succinic acid-producing strains in apple mash media. Lactic acid-producing strains (**a**) and succinic acid-producing strains (**b**). Apple mash (dark gray bar), apple mash supplemented with 5 g L^−1^ yeast extract (striped bar), and control medium (light gray bar). The error bars represent standard deviations

The screening of microorganisms based on OD measurements provides a preliminary assessment of the ability of a substrate to support microbial growth, as well as the adaptability of different strains to it, as reflected in biomass production [23]. The maximum OD values ranged from 0.173 to 0.988 for LA-producing strains and from 0.037 to 0.603 for SA-producing strains (Fig. 1). Based on the maximum OD relative to that of the control medium (Fig. 1) and the performance reported in previous studies [21], *H. coagulans* strains A35, A138, and A203 were selected for screening in bioreactors, and *A. succinogenes* DSM 22257 was chosen for subsequent SA fermentations. Notably, the superiority of *A. succinogenes* over *B. succiniproducens* in terms of SA production has already been reported [23, 24].

### Screening of microorganisms in bioreactors

**Fig. 2:**
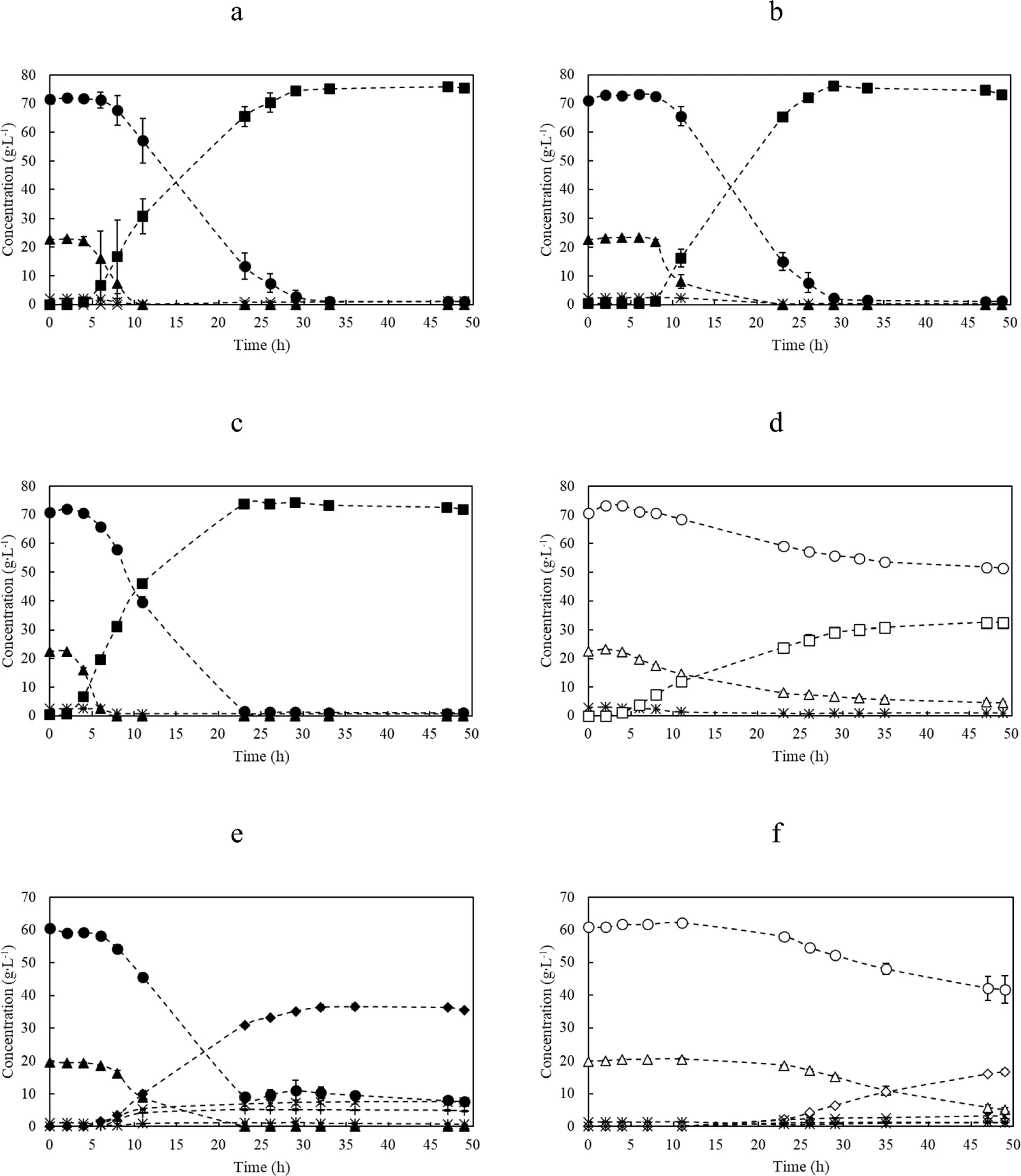
Sugar consumption and product formation in apple mash fermentations. 10 g L^−1^ yeast extract (solid symbols) and no yeast extract (open symbols). *H*. *coagulans* A35 (**a**), A138 (**b**), A203 (**c**,** d**), and *A. succinogenes* DSM 22257 (**e**,** f**). Fructose + malic acid (circle), glucose (triangle), sucrose (asterisk), lactic acid (square), succinic acid (diamond), acetic acid (x), and formic acid (+). The error bars represent standard deviations

The screening of microorganisms in bioreactors enables the identification of the best-performing strain under actual fermentation conditions, ensuring high yields and productivities and, consequently, a more effective process [20, 21]. The fermentation performance of LA strains selected in the initial OD-based assessment is illustrated in Fig. 2a-c, which shows that LA production ceased between 20 and 30 h when nearly all the sugars had been consumed.

LA production ranged from 71.8 to 75.5 g L^−1^, with yields varying between 0.91 and 0.94 g g^−1^ (Table 2). No significant differences were observed in LA concentration or yield among the strains (*p* > 0.05). However, strain A203 exhibited higher productivity, reaching 3.5 g L^−1^ h^−1^, compared with 2.5 g L^−1^ h^−1^ for strains A35 and A138. Additionally, longer lag phases were observed for strains A35 and A138 (Fig. 2a-c).

**Table 2:** Lactic and succinic acid production in apple mash fermentations by *H. coagulans* and *A. succinogenes* strains

Strain	Product (g L^−1^)	Yield (g g^−1^)	Productivity (g L^−1^ h^−1^)
*H. coagulans* A35	75.5 ± 0.2	0.94 ± 0.01	2.5 ± 0.1
*H. coagulans* A138	73.0 ± 1.5	0.92 ± 0.03	2.5 ± 0.1
*H. coagulans* A203	71.8 ± 0.2	0.91 ± 0.01	3.5 ± 0.0
*H. coagulans* A203 (no yeast extract)	32.3 ± 0.1	0.83 ± 0.06	0.7 ± 0.0
*A. succinogenes* DSM 22257	35.6 ± 0.3	0.69 ± 0.02	1.1 ± 0.1
*A. succinogenes* DSM 22257 (no yeast extract)	16.7 ± 0.8	0.70 ± 0.15	0.3 ± 0.0

The values represent the mean ± standard deviation

*H. coagulans* A203 has been evaluated in previous studies. When fermenting defatted rice bran hydrolysate, strain A203 produced 71.2 g L^−1^ LA, with a yield of 0.88 g g^−1^ and a productivity of 2.97 g L^−1^ h^−1^ [21]. It also demonstrated the ability to utilize pasta waste hydrolysate, producing approximately 44 g L^−1^ LA from 55 g L^−1^ sugars [20]. These findings highlight the efficiency of strain A203 in utilizing diverse substrates and further reinforce *H. coagulans* as a promising candidate for the biotechnological production of LA. Given its high LA concentration, yield, productivity, and strong performance in previous studies, strain A203 was selected for pilot-scale LA production using AM.

Following strain selection, the need for nutrient supplementation was evaluated (Fig. 2c and d). After 49 h of fermentation without the addition of yeast extract, only partial sugar consumption was observed, resulting in 32.3 g L^−1^ LA, and the productivity decreased to 0.7 g L^−1^ h^−1^. The influence of yeast extract on SA fermentation was also assessed using *A. succinogenes* DSM 22257, as shown in Fig. 2e and f. The absence of supplementation led to significant decreases in the SA concentration and productivity, from 35.6 g L^−1^ and 1.1 g L^−1^ h^−1^ to 6.7 g L^−1^ and 0.3 g L^−1^ h^−1^, respectively. Comparative values are presented in Table 2.

These results suggest that AM lacks sufficient nutrients to meet the demands of *H. coagulans* A203 and *A. succinogenes* DSM 22257 during fermentation, indicating the need for supplementation. Notably, higher ODs were observed in supplemented media in the preliminary strain screening (Fig. 1). Because nutrient supply is essential for AM fermentation, cost-effective alternatives to yeast extract are promising for reducing overall production costs [11]. Alternative nitrogen sources, such as wine lees, urea, and ammonium chloride, have been evaluated in apple pomace fermentations for LA production. Moreover, the use of agro-industrial by-products, such as tomato pomace, has shown potential to enhance fermentation performance, surpassing that achieved with yeast extract [14].

### Pilot-scale lactic acid fermentation

**Fig. 3:**
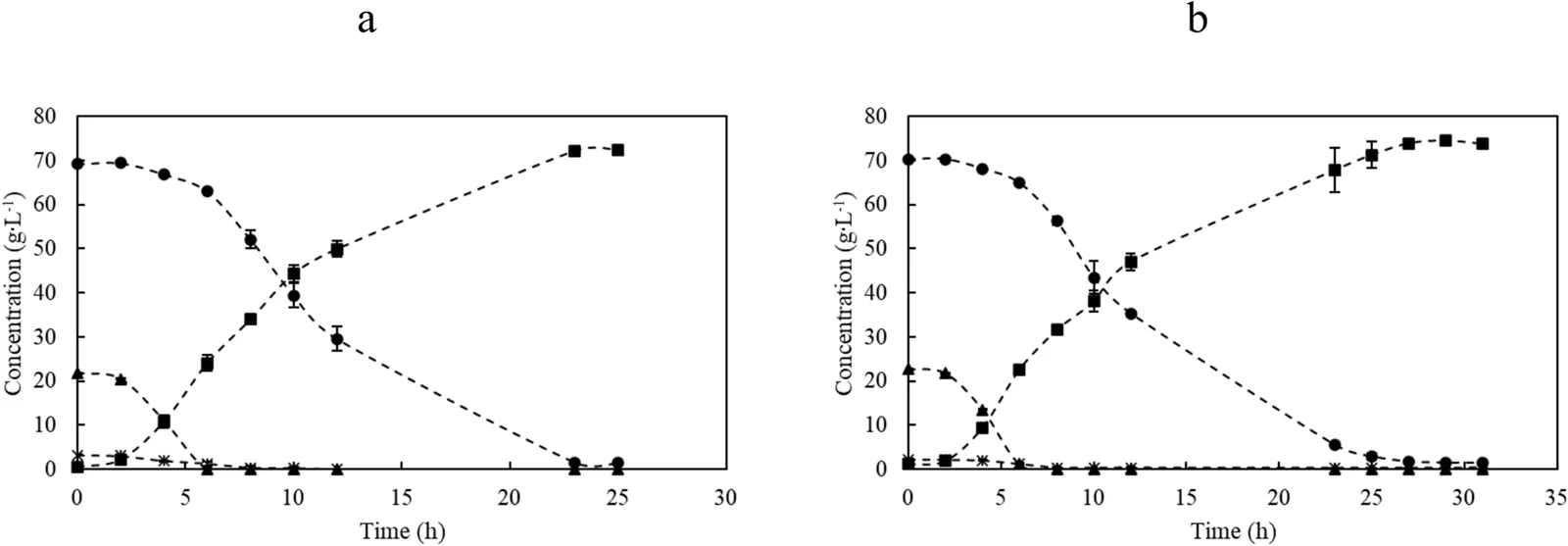
Lactic acid fermentation of apple mash by *H. coagulans* A203 at the laboratory and pilot scales. Laboratory scale (**a**) and pilot scale (**b**). Fructose + malic acid (filled circle), glucose (filled triangle), sucrose (asterisk), and lactic acid (filled square). The error bars represent standard deviations

The implementation of a laboratory-scale validation step is important to ensure that process simplifications do not compromise process stability, increase contamination risk, or reduce product yield prior to progressing to pilot-scale application [29]. The progress of the fermentation conducted in the prepilot-scale validation trial, in which the AM, following viscosity reduction, was directly supplemented and inoculated, is shown in Fig. 3a.

LA production reached 72.4 g L^−1^, with a yield of 0.93 g g^−1^ and a productivity of 3.4 g L^−1^ h^−1^ (Table 3). No significant differences in LA concentration, yield, or productivity were observed compared with the fermentation conducted in the previous step (Sect. "Screening of microorganisms in bioreactors") (*p* > 0.05), indicating that the applied method maintained process performance. The elimination of the sterilization step was achievable because of the elevated temperatures tolerated by the thermophilic strain *H. coagulans* employed in this study, which reduced the risk of contamination commonly associated with mesophilic microorganisms [11, 14]. The combination of a high fermentation temperature and a relatively short process duration further restricted the growth of potential contaminants [11]. Furthermore, although solid particles remaining after hydrolysis can negatively affect fermentation performance by causing inhibitory effects, mass transfer limitations, and operational issues in the bioreactor [30], the results indicate that centrifugation was not necessary in this process. Additional processing steps, such as centrifugation and sterilization between hydrolysis and fermentation, can significantly increase production costs and reduce the economic competitiveness of bioproducts compared with petrochemical alternatives, particularly at larger scales [20]; therefore, these results highlight the potential of the implemented procedure for efficient and cost-effective LA production.

**Table 3:** Lactic acid production in apple mash fermentations by *H. coagulans* A203 at the laboratory and pilot scales

Scale	Lactic acid (g L^−1^)	Yield (g g^−1^)	Productivity (g L-1 h^−1^)
Laboratory scale	72.4 ± 0.0	0.93 ± 0.01	3.4 ± 0.3
Pilot scale	73.8 ± 0.1	0.91 ± 0.00	2.7 ± 0.1

The values represent the mean ± standard deviation

Considering the outcomes of the process implemented at the laboratory scale, LA production from AM was scaled up (Fig. 3b). The analysis revealed that malic acid, which is naturally present in AM, was fully consumed by strain A203. Although a minor signal remained at the fructose retention time after fermentation, it was most likely caused by naturally occurring compounds in the AM that are not metabolized by the microorganism and co-elute with fructose under the HPLC conditions used, rather than residual fructose. The pilot-scale process achieved 73.8 g L^−1^ LA, a yield of 0.91 g g^−1^, and a productivity of 2.7 g L^−1^ h^−1^ (Table 3). The overall product yield was 0.78 g LA g^−1^ AM (dry basis). Notably, the final product exhibited an optical purity of 99.7% L-LA, indicating a highly selective fermentation and suggesting the absence of significant contamination, as contaminating bacteria typically produce both L- and D-isomers [11]. Although there were no significant differences in LA concentration, yield, or productivity between the laboratory and pilot scales, scaling up can often lead to lower productivities due to differences in mixing efficiency and limitations in heat and mass transfer, which can result in gradients in pH, temperature, and nutrients, thus affecting microbial behavior [31]. Moreover, the low standard deviations observed between biological replicates demonstrate the stability and reproducibility of the scaled-up process.

LA production from apple-derived feedstock has focused primarily on the use of apple pomace at the laboratory scale. The fermentation of apple pomace hydrolysate by *H. coagulans* DSM 2314 resulted in 40.72 g L^−1^ LA, with a yield of 0.96 g g^−1^ and a productivity of 0.58 g L^−1^ h^−1^ [14]. The superior outcomes obtained with AM (Table 3) are likely due to its higher sugar availability and lower content of inhibitory compounds, which are typically formed during lignocellulose pretreatment, promoting more efficient fermentation and increasing both the product concentration and the fermentation rate.

Although pilot-scale studies using apple residues are currently lacking, similar processes have been explored with other types of food waste. Fermentation of mixed food waste composed of fruits, vegetables, carbohydrates, meat, and dairy products resulted in 68.5 g L^−1^ LA, with a yield of 0.38 g g^−1^ total solids and a productivity of 0.95 g L^−1^ h^−1^ [17]. The utilization of pasta waste hydrolysate by *H. coagulans* A559 produced 47.67 g L^−1^ LA, achieving a yield of 0.67 g g^−1^ dry pasta waste. However, during upscaling, a 10-h increase in the lag phase was attributed to residual solid particles remaining after hydrolysis [20], an issue not encountered during the scale-up of AM fermentation. The higher product yield obtained with AM (0.78 g g^−1^) compared with both mixed food waste (0.38 g g^−1^) and pasta waste (0.67 g g^−1^) is likely attributable to several characteristics of the substrate. The compositional uniformity of AM enables more stable and reproducible fermentation than heterogeneous mixed food waste. Additionally, starch-rich materials, such as pasta waste, often require enzymatic hydrolysis and are associated with high viscosity, mass transfer limitations, and increased process complexity [11, 20]. Therefore, the enhanced performance and simplified operation observed in this study demonstrate the potential of waste apples as a viable substrate for effective and sustainable LA production.

### Pilot-scale succinic acid fermentation

**Fig. 4:**
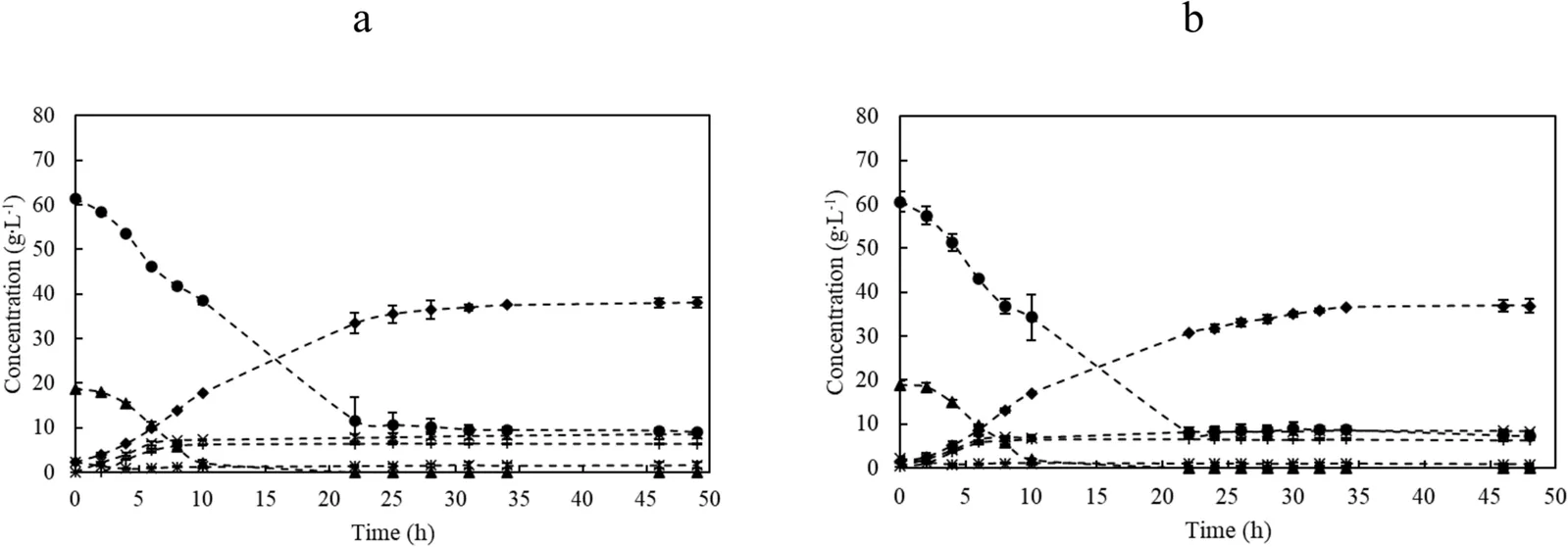
Succinic acid fermentation of apple mash by *A. succinogenes* DSM 22257 at the laboratory and pilot scales. Laboratory scale (**a**) and pilot scale (**b**). Fructose + malic acid (filled circle), glucose (filled triangle), sucrose (asterisk), succinic acid (filled diamond), acetic acid (x), and formic acid (+). The error bars represent standard deviations

Unlike *H. coagulans*, *A. succinogenes* grows optimally at 37–39 °C [12], a range that also favors the proliferation of competing microorganisms, which can negatively impact fermentation performance and outcomes [32]. In this study, microbial contamination was controlled by introducing a simplified thermal inactivation step (80 °C) following enzymatic liquefaction, and the performance during the subsequent fermentation at the laboratory scale is presented in Fig. 4a.

Compared with LA fermentation, SA fermentation began with a lower initial sugar concentration due to dilution from the addition of nutrient solution and inoculum. Consequently, the fermentation resulted in 38.1 g L^−1^ SA, with a yield of 0.69 g g^−1^ and a productivity of 1.1 g L^−1^ h^−1^ (Table 4). Acetic and formic acids were also detected at final concentrations of 8.5 g L^−1^ and 6.3 g L^−1^, respectively. No significant differences in SA concentration, yield, or productivity were observed compared with fermentation conducted with centrifuged and autoclaved medium, demonstrating the efficacy of the implemented procedure, which included the inactivation step. This is because most contaminating microorganisms cannot survive prolonged exposure to 80 °C, as this temperature exceeds the thermal limits of their cellular structures and enzymes, leading to protein denaturation and membrane disruption [33]. In addition, inactivation at 80–85 °C is a common method to reduce the microbial load without the high energy demands and specialized equipment required for sterilization, which is typically conducted at 121 °C under pressure [20]. Moreover, the milder conditions contribute to the preservation of nutrient integrity [21]. The results also demonstrate that the exclusion of the centrifugation step did not affect fermentation outcomes.

**Table 4:** Succinic acid production in apple mash fermentations by *A. succinogenes* DSM 22257 at the laboratory and pilot scales

Scale	Succinic acid (g L^−1^)	Acetic acid (g L^−1^)	Formic acid (g L^−1^)	Yield (g g^−1^)	Productivity (g L^−1^ h^−1^)
Laboratory scale	38.1 ± 1.1	8.5 ± 1.0	6.3 ± 0.2	0.69 ± 0.01	1.1 ± 0.4
Pilot scale	36.8 ± 1.5	8.3 ± 0.3	6.4 ± 0.2	0.69 ± 0.02	1.0 ± 0.1

The values represent the mean ± standard deviation

Considering these findings, SA production from apple pomace was scaled up. Sugar consumption and product formation followed the same trends observed at the laboratory scale (Fig. 4b). Sugars were nearly fully consumed, resulting in 36.8 g L^−1^ SA with a yield of 0.69 g g^−1^ (Table 4), while the productivity reached 1.0 g L^−1^ h^−1^. Acetic acid and formic acid were also produced, with final concentrations of 8.3 g L^−1^ and 6.4 g L^−1^, respectively. Additionally, approximately 5.0–6.0 g L^−1^ of malic acid was formed, and no fructose remained in the fermentation broth. The overall product yield was 0.55 g SA g^−1^ AM (dry basis). No significant differences in LA, acetic acid, or formic acid concentrations, yield, or productivity were observed, indicating that the scale-up process had no negative impact on fermentation performance. Similar to the LA fermentation, the stability and reproducibility of this approach are substantiated by the low standard deviations between biological replicates.

Studies on SA production from apple-derived feedstock have involved mainly theoretical investigations, relying on process modeling and technoeconomic analyses of apple pomace utilization by *A. succinogenes*. SA production has been assessed using life cycle assessment (LCA) to identify environmental impacts and process bottlenecks [15], and biogas coproduction has been proposed as a promising strategy [16]. Like LA, the pilot-scale production of SA by *A. succinogenes* has been investigated using other food wastes. The utilization of oat pomace and acid whey resulted in 19.6 g L^−1^ SA, achieving a yield of 0.77 g g^−1^ and a productivity of 0.27 g L^−1^ h^−1^ [18]. The abundance of readily available simple sugars (fructose and glucose), combined with the high moisture content (88.9%), facilitates rapid microbial assimilation and faster product accumulation. Consequently, higher titers and productivities were achieved than those obtained when using oat pomace and acid whey, which constitute a more complex and recalcitrant substrate. Furthermore, the use of oat pomace and acid whey requires the combination of two distinct waste streams to formulate the fermentation medium, potentially increasing the complexity of substrate preparation and processing. In turn, 38.99 g L^−1^ SA was produced from industrial candy waste, with a yield of 0.70 g g^−1^ and a productivity of 1.26 g L^−1^ h^−1^ [19]. Achieving comparable SA titers, yields, and productivities to those obtained from a refined, sugar-rich feedstock such as industrial candy waste demonstrates that apple mash provides an efficient fermentation medium capable of supporting bacterial growth without the need for extensive pretreatment, further supporting the feasibility of the proposed process.

Overall, the findings provide valuable data for the scaling up and implementation of biotechnological strategies aimed at the valorization of agri-food waste within the framework of biorefineries. The growing field of intelligent biorefineries, which integrates artificial intelligence and machine learning for adaptive control, offers further optimization by continuously adjusting processes in response to feedstock variability. This enables more efficient resource management, improves process stability and yield, and reduces costs. Future trends include the development of genetically engineered microbial strains designed to increase fermentation rates and product diversity, as well as the development of sustainable systems that minimize waste and energy consumption [34].

In addition to these upstream innovations, efficient DSP is essential for recovering and purifying bioproducts, ensuring their transformation into commercially viable products. The upstream simplifications proposed in this study offer several advantages for DSP. By retaining the solids within the reactor, the process eliminates the intermediate solid–liquid separation between enzymatic liquefaction and fermentation. Instead, only a single solid–liquid separation step is required after fermentation, as microbial biomass must be removed regardless of the process configuration [35]. Furthermore, retaining the solids minimizes sugar losses and allows microorganisms to access additional fermentable sugars and nutrients through the action of endogenous enzymes [36, 37]. Avoiding intermediate autoclaving also reduces heat-induced degradation of sugars and proteins, thereby limiting the formation of undesirable by-products [21]. Consequently, the overall process is simplified, separation efficiency is improved, and DSP costs, which represent an economic bottleneck at larger production scales, are expected to decrease [35, 38].

Finally, the proposed methodology offers economic and environmental advantages that support its potential as a cost-effective and sustainable process. From an economic perspective, its viability is enhanced by the utilization of waste apples, which serve as a zero- or low-cost feedstock, replacing expensive commercial sugars and avoiding the costly pretreatment steps required for lignocellulosic biomass such as apple pomace [9, 11, 14]. In addition, omitting the centrifugation step reduces operational costs associated with electricity consumption, maintenance, and labor, while avoiding autoclaving at 121 °C—either by eliminating it or replacing it with thermal inactivation—reduces the energy required for steam generation and subsequent bioreactor cooling. From an environmental perspective, these process simplifications reduce energy consumption, thereby lowering the carbon footprint and associated CO_2_ emissions [20, 37]. Water consumption is also reduced because the high moisture content of the AM (88.9%) minimizes water input. In the case of SA production, the metabolic pathway of *A. succinogenes* incorporates CO_2_ during succinate formation, further enhancing the environmental sustainability of the process [15, 19]. Lastly, the valorization of waste apples diverts organic waste from landfills, reducing the potential generation of methane and other greenhouse gases [37]. Together, these advancements highlight the potential of integrated biorefinery approaches that not only convert agri-food residues into high-value bioproducts but also contribute to a more circular and sustainable bioeconomy.

## Conclusions

Pilot-scale production of LA and SA from waste apples was successfully demonstrated. Preliminary screening identified *H. coagulans* A203 and *A. succinogenes* DSM 22257 as the most suitable candidates for LA and SA production, respectively, when apple mash was used as the substrate. An alternative fermentation procedure confirmed the feasibility of conducting the process without costly centrifugation and sterilization steps. Pilot-scale LA production resulted in 73.8 g L^−1^ LA, a yield of 0.91 g g^−1^ and a productivity of 2.7 g L^−1^ h^−1^. For SA, 36.8 g L^−1^ was achieved, corresponding to a yield of 0.69 g g^−1^ and a productivity of 1.0 g L^−1^ h^−1^. These results validate the potential of waste apples as a feedstock for LA and SA production, covering microorganism selection, process development, and pilot-scale fermentation. Overall, this study supports the integration of agri-food residues into biotechnological processes aimed at generating high-value products, contributing to the ongoing efforts to implement a circular and sustainable bioeconomy. Moving forward, continued advancements in microbial strain development and process optimization will be crucial for enhancing product yields and achieving scalable, cost-effective production, further promoting biorefinery approaches.

## Supplementary Information

Supplementary Material 1

## Data Availability

All data generated or analyzed during this study are included in this published article.

## Abbreviations

AM: Apple mash
DSP: Downstream processing
LA: Lactic acid
mL_e_ kg_bm_^−^^1^: Milliliters of enzyme per kilogram of biomass
SA: Succinic acid
