# Supplementary material for: Upcycling waste apples into platform chemicals: pilot-scale production of lactic and succinic acids
Source: Biotechnol Biofuels Bioprod. 2026 Sep 7;19:67. doi: 10.1186/s13068-026-02807-w (PMC13548481; doi:10.1186/s13068-026-02807-w)
Supplement: Supplementary file 1 [file 13068_2026_2807_MOESM1_ESM.docx]

| a   | b   |
| --- | --- |

**Figure S1.** Waste apples before mechanical pressing (a). Apple mash obtained after mechanical pressing of the waste apples (b).
